# Cholinergic and inflammatory phenotypes in transgenic tau mouse models of Alzheimer’s disease and frontotemporal lobar degeneration

**DOI:** 10.1093/braincomms/fcaa033

**Published:** 2020-03-30

**Authors:** Anna L Cranston, Adrianna Wysocka, Marta Steczkowska, Maciej Zadrożny, Ewelina Palasz, Charles R Harrington, Franz Theuring, Claude M Wischik, Gernot Riedel, Grazyna Niewiadomska

**Affiliations:** f1 School of Medicine, Medical Sciences and Nutrition, University of Aberdeen, Foresterhill, Aberdeen AB25 2ZD, UK; f2 Nencki Institute of Experimental Biology, Polish Academy of Science, Warsaw 02-093, Poland; f3 Mossakowski Medical Research Centre, Warsaw 02-106, Poland; f4 TauRx Therapeutics Ltd, Foresterhill, Aberdeen AB25 2ZP, UK; f5 Institute of Pharmacology, Charite—Universitätsmedizin Berlin, Berlin, Germany; f6 TauRx Therapeutics Ltd, Aberdeen AB24 5RP, UK

**Keywords:** Alzheimer’s, cholinergic, tauopathy, neuroinflammation, tau

## Abstract

An early and sizeable loss of basal forebrain cholinergic neurons is a well-characterized feature associated with measurable deficits in spatial learning and cognitive impairment in patients with Alzheimer’s disease. In addition, pro-inflammatory glial cells such as astrocytes and microglia may play a key role in the neurodegenerative cascade of Alzheimer’s disease and tauopathies. We recently presented two mouse models: Line 1, expressing the truncated tau fragment identified as the core of the Alzheimer’s paired helical filament, and Line 66, expressing full-length human tau carrying a double mutation (P301S and G335D). Line 1 mice have a pathology that is akin to Alzheimer’s, whilst Line 66 resembles frontotemporal lobar degeneration. However, their cholinergic and inflammatory phenotypes remain elusive. We performed histological evaluation of choline acetyltransferase, acetylcholinesterase, p75 neurotrophin receptor, microglial ionized calcium binding adaptor molecule 1 and astrocytic glial fibrillary acidic protein in the basal forebrain, hippocampus and cortex of these models. A significant lowering of choline acetyltransferase-positive neurons and p75-positive neurons in the basal forebrain of Line 1 at 3, 6 and 9 months was observed in two independent studies, alongside a significant decrease in acetylcholinesterase staining in the cortex and hippocampus. The reductions in choline acetyltransferase positivity varied between 30% and 50% at an age when Line 1 mice show spatial learning impairments. Furthermore, an increase in microglial ionized calcium binding adaptor molecule 1 staining was observed in the basal forebrain, hippocampus and entorhinal cortex of Line 1 at 6 months. Line 66 mice displayed an intact cholinergic basal forebrain, and no difference in p75-positive neurons at 3 or 9 months. In addition, Line 66 exhibited significant microglial ionized calcium binding adaptor molecule 1 increase in the basal forebrain and hippocampus, suggesting a prominent neuroinflammatory profile. Increased concentrations of microglial interleukin-1β and astrocytic complement 3 were also seen in the hippocampus of both Line 1 and Line 66. The cholinergic deficit in Line 1 mice confirms the Alzheimer’s disease-like phenotype in Line 1 mice, whilst Line 66 revealed no measurable change in total cholinergic expression, a phenotypic trait of frontotemporal lobar degeneration. These two transgenic lines are therefore suitable for discriminating mechanistic underpinnings between the Alzheimer’s and frontotemporal lobar degeneration-like phenotypes of these mice.

## Teaser

We present here two tau transgenic models; L1 expressing a truncated tau fragment and L66 full-length, mutant human tau. A cholinergic deficit and region-specific inflammation in L1 mice confirms an Alzheimer's disease-like phenotype, whilst Line 66 revealed no measurable change in cholinergic expression, a phenotypic trait of frontotemporal lobar degeneration.

## Graphical Abstract

**Graphical Abstract:**
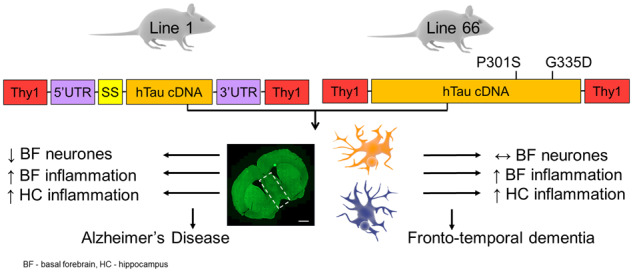

## Introduction

Tauopathies are a diverse group of neurodegenerative disorders associated with cognitive and behavioural impairments in the ageing population. The most prevalent, Alzheimer’s disease, is a complex and heterogeneous neurodegenerative disease of the CNS, first presented by German psychiatrist Alois Alzheimer (Alzheimer, 1907), typically characterized by the deposition of extracellular β-amyloid plaques and intracellular tau neurofibrillary tangles (NFTs). Tau is a low-molecular-weight microtubule-associated protein, expressed predominantly in axons (Buee *et al.*, 2000) and also dendrites (Ittner and Ittner, 2018). Tau regulates the assembly and stability of microtubules, and its microtubule-binding function is regulated negatively through phosphorylation (Weingarten *et al.*, 1975). In Alzheimer’s disease, tau accumulates as NFTs and ultimately into paired helical filaments. The core of the paired helical filament is composed of a truncated 95-amino-acid fragment of tau (Wischik *et al.*, 1988) and is capable of catalytic conversion of normal soluble tau into intracellular oligomeric aggregates (Wischik *et al.*, 1995, 1996), resulting in dystrophic neurites, dendrites and cell bodies, a characteristic pathology of tauopathies (Spires-Jones *et al.*, 2009). The role of phosphorylation in this process remains unknown (Kimura *et al.*, 2018).

A significant loss of up to 50% of basal forebrain cholinergic neurons is another well-characterized feature of Alzheimer’s disease (Davies and Maloney, 1976; Perry *et al.*, 1985). Acetylcholine plays a crucial role in the peripheral nervous system and central nervous system and can be measured by the presence of neuronal choline acetyltransferase (ChAT; Nagai *et al.*, 1983). In addition, an age-dependent decline in acetylcholinesterase (AChE), the enzyme responsible for acetylcholine hydrolysis (Ferreira-Vieira *et al.*, 2016), is associated with reduced AChE in patients with Alzheimer’s disease (Hirano *et al.*, 2012; Janeczek *et al.*, 2018). As such, current symptomatic Alzheimer’s disease treatments, specifically AChE inhibitors donepezil and rivastigmine, act to remediate this cholinergic dysfunction through increasing the level of acetylcholine at the synaptic cleft (Raina *et al.*, 2008).

Intriguingly, cholinergic degeneration is lacking in frontotemporal lobar degeneration (FTLD), a progressive neurodegenerative tauopathy primarily affecting the frontal and temporal lobes of the brain (Wilkinson *et al.*, 2002; Roy *et al.*, 2016). This has further strengthened the argument favourably towards the ‘Cholinergic Hypothesis’ of Alzheimer’s disease (Terry and Buccafusco, 2003) and is supported by histological post-mortem studies showing that low-affinity p75 neurotrophin receptor (p75^NTR^), a marker of cholinergic basal forebrain neurons, is significantly reduced in mild cognitive impairment (Mufson *et al.*, 2002). Therefore, an in-depth understanding of the cholinergic system is key to deciphering the pathological dissociation between Alzheimer’s disease and other tauopathies. The basal forebrain cholinergic system is composed of the medial septum (MS), horizontal limb of the diagonal band of Broca (HDB) and vertical limb of the diagonal band of Broca (VDB), nucleus basalis of Meynert (classified as the nucleus basalis magnocellularis in the murine brain; nBM), nucleus accumbens and the substantia innominata (Niewiadomska *et al.*, 2009). As a network, these regions provide the major cholinergic projections via the septo-hippocampal pathway to the entorhinal cortex (EC) and hippocampus, regions that exhibit parallel tau pathology (Niewiadomska *et al.*, 2006). Afferent cholinergic projections from the MS to hippocampus have been shown as a critical pathway in mediating spatial and mnemonic function (Robinson *et al.*, 2010; Deiana *et al*; 2011; Okada *et al.*, 2015).

Several tau transgenic (Tg) mouse models have been generated (Götz *et al.*, 2001; Yanamandra *et al.*, 2015) to explain the pathogenic mechanisms of tau and to assess the efficacy of therapeutic targets for Alzheimer’s disease and FTLD. Familial autosomal dominantly inherited *MAPT* mutations provide a basis for the majority of tau Tg mouse models generated to date. These include a tau Tg mouse that overexpresses tau carrying the mutation P301L (Götz *et al.*, 2001), a mutation associated with FTLD (Caillet-Boudin *et al.*, 2015). Overexpression of human P301L tau in cortical and hippocampal neurons increases astrocytosis in addition to neuronal apoptosis, similar to those observed in human tauopathies (Götz *et al.*, 2001). Alternative approaches to the P301L model have explored the overexpression of human wild-type (WT) tau in mice, observing synapse loss and microglial activation, in some cases even preceding the deposition of intracellular NFTs (Yoshiyama *et al.*, 2007). A limitation of the P301L model is that these animals exhibit motor deficits and hind limb paralysis, traits that are not consistent with symptoms observed in patients with Alzheimer’s disease. Also, microglial activation without the appearance of NFT pathology can frequently be observed in these mice (Götz *et al.*, 2001), presenting a model lacking true translational features.

The cholinergic Alzheimer’s disease-like dysfunction has been characterized for several Tg rodent tau models, most recently in the THY-Tau22 mouse, a model that displayed major hippocampal Alzheimer’s disease-like tau pathology within the septo-hippocampal pathway (Belarbi *et al.*, 2009). This model also presented a significant reduction in ChAT-immunoreactive neurons in the MS. Furthermore, an age-dependant mild astrogliosis was reported in THY-Tau22 mice, particularly in association with tau-positive cells (Schindowski *et al.*, 2006).

A pro-inflammatory activation of microglia and astrocytes has recently been identified to play a key role in acting as secondary effector in neurodegenerative cascades in the human brain, with neuroinflammation considered to precede the onset of both Alzheimer’s disease and FTLD symptoms in patients (Heneka *et al.*, 2014; Morales *et al.*, 2014). This is consistent with observations in P301L tau Tg mice (Götz *et* al., 2001). In patients with Alzheimer’s disease, a strong correlation exists between NFT deposition and density of activated microglia, typically established through immunohistochemical staining of the ionized calcium binding adaptor molecule 1 (Iba1), a robust marker of neuroinflammation (Arends *et al.*, 2000). In addition, increased inflammatory mediators, such as tumour necrosis factor alpha and interleukin-1 beta (IL-1β), are correlated with cognitive deficits for patients with late-stage Alzheimer’s disease (Morimoto *et al.*, 2011). In particular, sustained IL-1β overexpression secreted by activated microglia is known to exacerbate tau pathology in tau-overexpressing mice (Ghosh *et al.*, 2013) and up-regulation of complement C3 protein, a marker of activated A1 astrocytes, has been observed in PS19 mice in response to NFT pathology (Litvinchuk *et al.*, 2017).

Current animal models of tauopathy and Alzheimer’s disease fail to address the specific molecular mechanisms by which products of inflammation produced through activated microglia and astrocytes can induce Alzheimer’s disease neurodegeneration and the potential link between the observed cholinergic basal forebrain degeneration. Therefore, we conducted a robust evaluation of the cholinergic system of Line 1 (L1) and Line 66 (L66) Tg mice to decipher the phenotypic differences in these two genetically distinct models. Given the pathological and behavioural phenotypes previously described in these models (Melis *et al.*, 2015), the cholinergic phenotypes were performed in two independent studies undertaken in Warsaw, Poland, and Aberdeen, UK, alongside a robust evaluation of the neuroinflammatory phenotype of these two Tg mouse models.

## Materials and Methods

### Tg animals

Female homozygous Tg L1, L66 and WT NMRI litters were generated as previously described (Melis *et al.*, 2015). All animals were bred commercially at Charles River, UK, and distributed to the respective facilities (road for Aberdeen, air-freight for Warsaw) 1 month prior to use to allow ample acclimatization. Animals were colony housed (up to four per cage) in wire-lid cages (Makrolon II) on corn cob bedding and enrichment (paper strips and cardboard tubes) in a controlled facility (temperature 20–22°C, 60–65% humidity, air changes: 17–20 changes per hour) and ad libitum access to water and food pellets and under a 12-h light/dark cycle (lights on at 07:00 am). Experiments were carried out in accordance with the European Communities Council Directive (63/2010/EU), a project licence with local ethical approval under the UK Animals (Scientific Procedures) Act (1986), or with approval from First Warsaw Local Ethics Committee for Animal Experimentation, and carried out in accordance with Polish Law on the Protection of Animals and National Institute of Health’s Guide for Care and Use of Laboratory Animals (Publication No. 85-23, revised 1985).

### Immunohistochemistry

Brains were processed independently from mice aged 3, 9 and 12 months (±2 weeks) in Warsaw, Poland, and at 6 months (±2 weeks) in Aberdeen, UK. Immunohistochemical analyses were carried out in independent studies in Aberdeen, UK [ChAT, Iba1, glial fibrillary acidic protein (GFAP)], and Warsaw, Poland (ChAT, p75^NTR^, AChE).

#### Fluorescent immunohistochemistry for ChAT, Iba1 and GFAP (Aberdeen)

Adult female homozygous 6-month-old Tg L1, L66 and WT NMRI mice were randomly selected for terminal intraperitoneal injection of pentobarbital (Euthatal; 0.2 ml/30 g body weight; Merial Animal Health Ltd, Lyon, France). Anaesthetized mice were transcardially perfused with 0.9% (w/v) saline, followed by 4% paraformaldehyde (Sigma-Aldrich, UK) until fixation was achieved. Skulls were immediately dissected, and whole brains removed and post-fixed in 4% paraformaldehyde (4 h), followed by 24 h incubation in 10% sucrose (Phosphate buffered saline (PBS); pH = 7.4), 30% sucrose (PBS) and 2% DMSO/2% glycerol (Sigma-Aldrich) at 4°C over 3 days. Following post-fixation, brains were snap-frozen in isopentane (2-methylbutane; Sigma-Aldrich).

Free-floating sections were used from whole brains embedded in OCT matrix (CellPath Ltd, Newtown, UK). Coronal sections (30 µm) were obtained (CM1850 Cryostat; Leica Biosystems) from brain regions of interest [‘Bregma 1.4’; ‘Bregma −3.64 mm’, according to Paxinos and Franklin (2001)] and stored in cryoprotectant solution (glycerol, ethylene glycol, 0.1 M PB, 1:1:2) until staining. Every second section (nBM, VDB) or every third section [MS, HDB, striatum (ST), EC, CA1, hilus, granular layer, sub-granular zone (SGZ) and molecular layer of dentate gyrus (DG)] was stained (3× sections in total). Sections were blocked in 1% milk powder, 2% bovine serum albumin (BSA), 0.3% Triton X-II, 1.5% normal goat serum (Chemicon^®^) or 5% normal donkey serum (Sigma-Aldrich) for 1 h at room temperature (RT), rocking. Followed by 3 × 5 min PBS washes, sections were incubated in primary antibody, diluted in PBS containing 2% BSA, 0.3% Triton X-II, 1.5% normal goat serum or 5% normal donkey serum to working concentrations (Supplementary Table 1) overnight at 4°C, and the following day for 1 h at RT. Sections were washed (3 × 5 min, PBS) before incubation (1 h, RT) with secondary antibody, containing 2% BSA and PBS, diluted to working concentrations. Serial sections were mounted onto slides (VWR^®^ SuperFrost Microscope Slides, 1-mm thick; Borosilicate glass coverslips, 22 mm × 50 mm, VWR^®^ International), using ProLong Gold Antifade Mountant with 4′,6-diamidino-2-phenylindole (Thermo Fisher Scientific, UK) for nuclear staining.

#### HRP-enhanced immunohistochemistry of ChAT and p75^NTR^ (Warsaw)

Female, homozygous L1, L66 and WT (NMRI) mice at 3 and 9 months were terminally anaesthetized with pentobarbital (Morbital; 1.7 ml/kg) by intraperitoneal injection. Mice were subsequently perfused with 0.01 M PBSV with 0.05% heparin (w/v) and then with 4% paraformaldehyde containing 15% saturated picric acid for fixation and cryopreserved in 5% glycerol/2% DMSO (0.1 M PBSV, pH = 7.4). Whole brains were dissected and post-fixed in 4% paraformaldehyde (12 h), followed by a 24 h incubation in 10% glycerol/2% DMSO (0.1 M PBS, pH = 7.4; 1 mM Na_3_VO_4_; PBSV) and 20% glycerol/2% DMSO (PBSV), over 3 days. After post-fixation, brains were snap-frozen on dry ice and stored in −80°C until staining. Whole brains were embedded in Shandon™ Cryomatrix™ embedding resin (Thermo Fisher Scientific, USA) and sectioned at 40 µm. Free-floating sections were washed in 0.1 M PBSV (3 × 5 min) and incubated with 1% H_2_O_2_ (Chempur, Poland) for 30 min to block endogenous peroxidases. Sections were blocked in a serum solution [5% normal rabbit serum (Vector Laboratories, USA) or 5% normal goat serum (Vector Laboratories, USA) (for ChAT and p75^NTR^, respectively), 0.3% Triton X-100 (Sigma-Aldrich, USA) in 0.1 M PBSV]. They were then incubated in primary antibody (Supplementary Table 1) in blocking solution (1% BSA) for 1 h at RT, and overnight at 4°C. Sections were washed in PBSV (3 × 5 min) before incubation with secondary antibody (0.1 M PBSV with 5% normal rabbit serum/5% normal goat serum, 1% BSA, 0.3% Triton X-100), diluted to working concentrations (Supplementary Table 1) for 1 h at RT. Sections were washed (PBSV, 3 × 5 min) and incubated with horseradish peroxidase-conjugated streptavidin, 1% BSA, and PBSV (1:500; Vector Laboratories Inc.). Following 3 × 5 min PBSV washes, sections were finally incubated with 3,3′-diaminobenzidine (0.025%) in 0.1 M PBSV containing 0.04% NiSO_4_ and 0.025% H_2_O_2_. The reaction was stopped via the addition of 0.1 M PB (pH 7.4). After 3 × 5 min washes (0.1 M PB), sections were mounted onto glass slides, allowed to dry, before immersion in xylene, and mounted in DePeX.

### Enzyme-linked immunosorbent assay of IL-1β and C3

Adult female homozygous 6-month-old L1, L66 Tg mice and WT NMRI mice were decapitated and the ST (with nucleus accumbens) and hippocampus collected and stored at −80°C, until further use. Concentrations of IL-1β (*n* = 3) and complement 3 (C3, *n* = 5) were quantified by enzyme-linked immunosorbent assay in regions of interest (Mouse Interleukin 1beta ELISA Kit, MyBioSource, USA; Mouse Complement Component 3 ELISA Kit, MyBioSource, USA), according to manufacturer’s instructions, and results expressed in pictograms per millilitre and micrograms per millilitre, respectively. All standards and samples were added in duplicate to the microtiter plate, and the IL-1β and C3 concentrations were calculated from standard curves.

### AChE histochemistry

In accordance with a modified version of Koelle–Friedenwald method (Koelle and Friedenwald, 1949), air-dried sections on glass slides were rinsed in double-distilled water and, to reveal hippocampal or cortex staining, incubated for 1 or 1.5 h, respectively, at RT, shaking, in solution (ethopropazine, glycine, CuSO_4_·5H_2_O, acetylthiocholine iodide, sodium acetate, glacial acetic acid, double-distilled water). Slides were washed 6 × 5 min with water and developed (1.25% sodium sulphide solution) for 1 min. Slides were washed again for 6 × 5 min with water and incubated with intensifying solution (1% AgNO_3,_ w/v) in water, washed 3 × 5min with water and incubated again for 2 ×5 min (5% Na_2_S_2_O_3_, w/v), at RT with shaking. Finally, slides were washed 6 × 5 min with water before immersion in xylene and mounted in DePeX.

### Quantitative analysis of ChAT, Iba1 and GFAP-IR neurons (Aberdeen)

Fluorescent images of ChAT, Iba1 and GFAP-IR were obtained under a Zeiss-Axio Imager M1 upright fluorescence microscope (Zeiss, Germany). Stereological cell counting of ChAT-stained neurons was recorded using the Optical Fractionator method of Stereo Investigator Stereology Software (MBF Bioscience). Regions were identified microscopically in accordance with the Mouse Brain Stereotaxic Atlas (Paxinos and Franklin, 2012) as MS, nBM the VDB and HDB, the nucleus basalis magnocellularis (nBM), ST and EC. Hippocampal regions were identified as the sub-regions of the DG: hilus, granular layer, SGZ molecular layer and as CA1 (see Supplementary Fig. 1). A cell was considered as a cholinergic neuron when the following criteria were reached: ChAT immunoreactivity of neuronal somata as well as good visibility of cell nucleus and proximal segment of two or more dendrites within the counting frame, allowing for the exclusion of neuronal debris. Regions were outlined using a 2.5× magnification, and cell counting was carried out using the 20× magnification by an experienced experimenter. There was no sample blinding.

### Quantitative analysis of ChAT-IR neurons (Warsaw)

Sections were imaged under a Nikon Eclipse Ni-E microscope. The computerized densitometric image analysis (NIS-Elements BR4.30.00; Nikon Instruments) of ChAT-IR neurons was performed in the basal forebrain regions (MS, VDB, HDB, nBM and ST). Regions were identified microscopically in accordance with the Mouse Brain Stereotaxic Atlas (Paxinos and Franklin, 2012). Regions of interest were outlined using the software’s X–Y plotting system that measures the square area (mm^2^) of the marked frame, and ChAT-IR neurons were counted manually at 400× magnification. Cell counts per section were then corrected with the Abercrombie’s formula (Abercrombie, 1946), and the packing density of the cholinergic neurons was calculated as a function of rostro-caudal level and location within the studied structures by using the obtained cell counts and the square area of the marked frames in each analysed section. Therefore, the mean number of ChAT-IR neurons obtained from each experimental group was expressed as packing density (the number of cells/mm^2^).

### Quantitative analysis of AChE

AChE was analysed microscopically at 100× magnification using NIS-Elements BR4.30.00 Software. Measurements were taken from a fixed area of 75 000 ± 500 mm^2^ in the primary somatosensory cortex and the CA3 hippocampal area, which comprises a cross-section of all cortical or hippocampal layers. Data are presented as the mean optical density in Tg L1 or L66 (for both lines *n* = 3) or WT mice (*n* = 2) at 3 and 12 months of age.

### Statistical analysis

All data sets are expressed as group mean ± SEM for the number of experiments indicated in the figure and table legends. Data were analysed using one-way or two-way ANOVA, followed by *post hoc* comparison using two-tailed Student’s *t*-tests and Newman–Keuls test. Specific weight was given to the comparison between Tg and WT mice, but not between Tg lines. The Mann–Whitney *U*-test for independent groups was also used, when appropriate. Differences between groups were considered statistically significant for *P < 0.05. Statistical analyses were performed using GraphPad Prism 5.04 (Aberdeen, UK) and STATISTICA 12 software (Warsaw, Poland).

### Data availability

All the raw data supporting the results of this study are available at the reader’s request.

## Results

### Loss of cholinergic neurons in the basal forebrain of L1 mice

**Figure 1:**
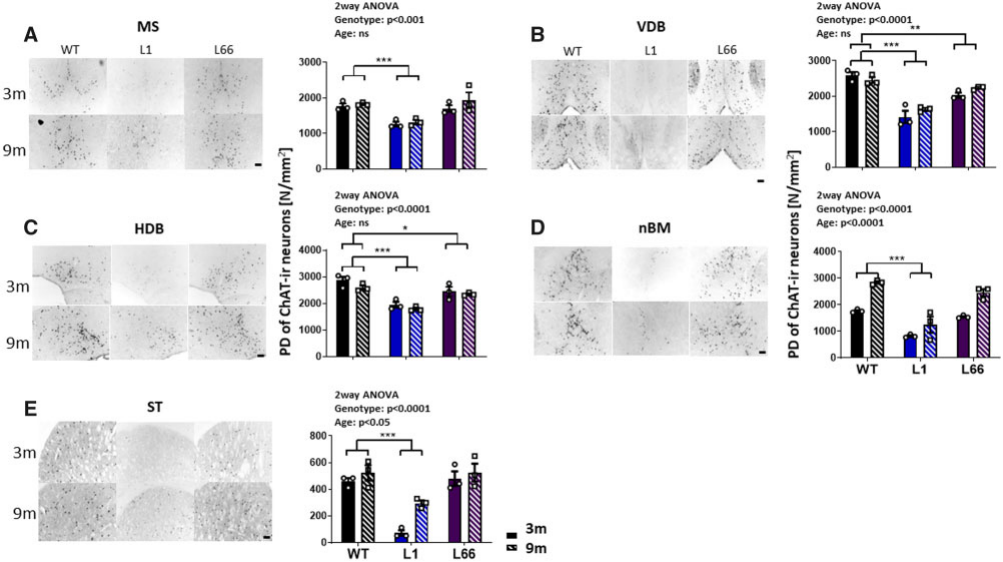
**ChAT staining intensity is reduced in L1 basal forebrain at 3 and 9 months.** Representative microphotographs and quantification of ChAT immunohistochemical staining in the basal forebrain regions of (**A**) MS, (**B**) VDB, (**C**) HDB, (**D**) nBM and (**E**) ST in 3- and 9-month-old transgenic L1 (*n* = 3), L66 (*n* = 3) and WT (NMRI; *n* = 3) mice. Images were obtained at a 100× magnification. Scale bar: 200 μm. Cell counts are expressed as PD of ChAT-IR neurons (N/mm^2^). The effect of genotype was determined using a two-way ANOVA, with transgene and age as factors followed by the Newman–Keuls test. Data were considered statistically significant with alpha set to 5%. *P < 0.05, **P < 0.01 and ***P < 0.001. Data presented as mean ± SEM. PD = packing density; ST = striatum.

**Figure 2:**
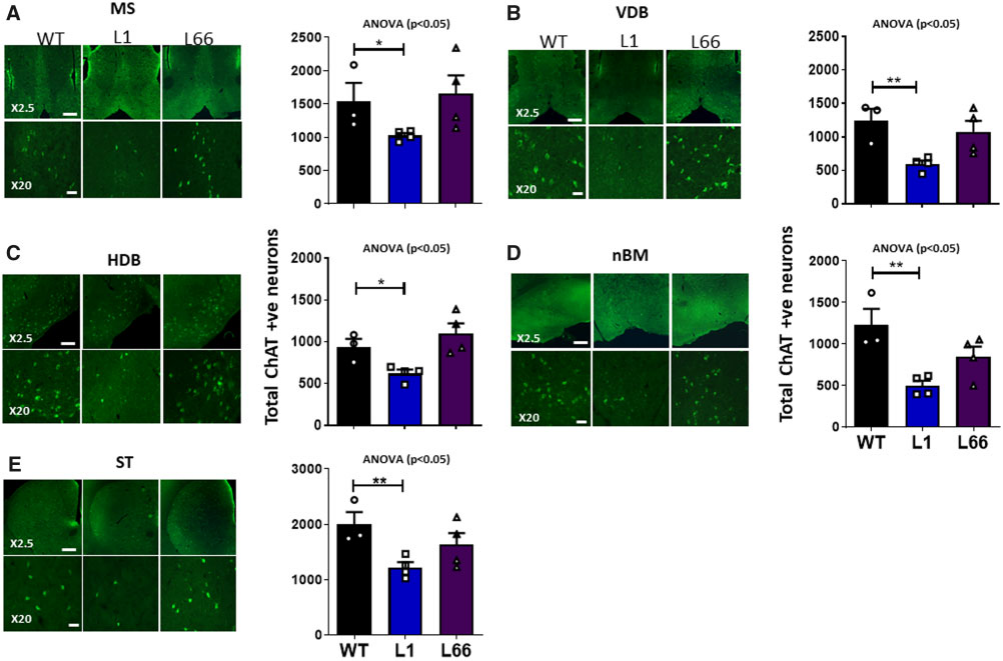
**ChAT staining intensity is reduced in L1 basal forebrain at 6 months.** Fluorescence microscopic images of ChAT expression and ChAT stereological cell counting from basal forebrain regions of the (**A**) MS, (**B**) VDB, (**C**) HDB, (**D**) nBM and (**E**) ST in L1 (*n* = 4), L66 (*n* = 4) and WT (NMRI, *n* = 3) mice. Representative images were obtained at 2.5× or 20× magnification, as indicated. Scale bar 2.5×, 450 µm; 20×, 55 µm. Cell counts are expressed as total cells. The effect of genotype was determined using a one-way ANOVA and individual genotype differences by Student’s *t*-test. Data were considered statistically significant when *P < 0.05 and **P < 0.01. Data presented as mean ± SEM. ST = striatum.

Immunofluorescence levels of the ChAT enzyme were determined using two independent experiments using two immuno-histochemical staining methods in coronal mouse brain sections of the basal forebrain. Sections from L1 animals at 3, 6 and 9 months show a clear reduction in both density and total ChAT-IR neuron cell count in basal forebrain regions of the MS, nBM, VDB, HDB and ST, compared to WT brain sections (Figs 1 and 2). A two-way ANOVA with genotype and age returned a *P* < 0.001 for the factor genotype in all regional comparisons. This effect was mainly due to significantly reduced ChAT-IR packing densities in basal forebrain and ST of L1 (Fig. 1A–E, MS, VDB, HDB, nBM and ST; all *P*-values <0.001 compared with WT). In addition, the age factor was significant only in nBM (*P* < 0.0001) and in ST (*P* < 0.05), but not in MS, VDB or HDB.

In agreement with the above profile, an independent set of experiments using fluorescence measured total ChAT cell counts in L1 and L66 at 6 months of age. It confirmed reduction in ChAT positivity in L1 for all regions of the basal forebrain, but not in L66. The reduction for all regions was between 30% (MS) and 60% (nBM) in L1 compared to WT (Fig. 2A–E, Supplementary Table 2).

### Altered AChE in cortex and hippocampus of L1 mice

**Figure 3:**
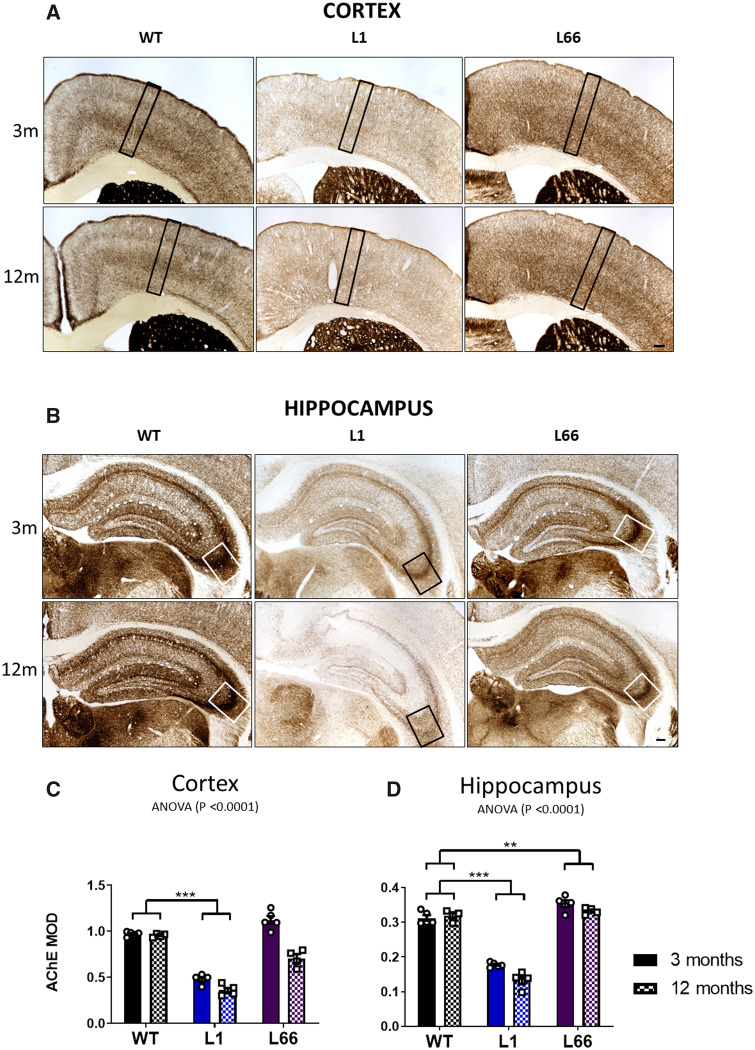
**Intensity of AChE staining is reduced in L1 cortex and hippocampus.** Representative microphotographs of AChE MOD in cortex (**A**) and hippocampus (**B**) of transgenic L1, L66 and WT (NMRI) mice at 3 and 12 months. *n* = 5 for all groups except WT, L66 cortex at 12 months, and WT, L66 hippocampus at 12 months, where *n* = 4 per group. The rectangle represents the area of measurement for the analysis of MOD in the cortex and hippocampus. Scale bar: 100 μm. MOD of AChE quantified in cortex (**C**) and hippocampus (**D**) of transgenic L1, L66 and WT (NMRI) mice at 3 and 12 months. The AChE is presented as MOD + SEM. Main effects were determined using two-way ANOVA and the source of significance explored by *post hoc* Newman–Keuls test. Data were considered statistically significant with alpha set to 5%. *P < 0.05, **P < 0.01 and ***P < 0.001. Data presented as mean ± SEM. MOD = mean optical density.

The AChE activity was assessed histochemically from an area cross-sectioning through all cortical and hippocampal layers. The staining was visibly weaker in L1 mice compared with WT at 3 and 12 months (Fig. 3). Densely stained individual cortical layers could not be distinguished in L1 tissue, whereas WT and L66 exhibited two distinct bands with high staining intensity corresponding with cortical layers II and V. This was confirmed quantitatively (Fig. 3C and D). There was also a significant decrease in AChE staining within cortex of L1 mice compared with age-matched WT (*P* < 0.001; Fig. 3C).

AChE staining in hippocampus was lower in L1 relative to WT (*P* < 0.001) (Fig. 3D). Conversely, increased AChE staining intensity was observed in L66 relative to WT (*P* < 0.01) probably due to the elevated AChE level in 3-month L66 mice as compared to age-matched WT. Moreover, L1 and L66 mice displayed a significant decrease in AChE staining with age (age factor *P* < 0.05) (Fig. 3D).

### Decreased low-affinity neurotrophin receptor staining in L1 basal forebrain

**Figure 4:**
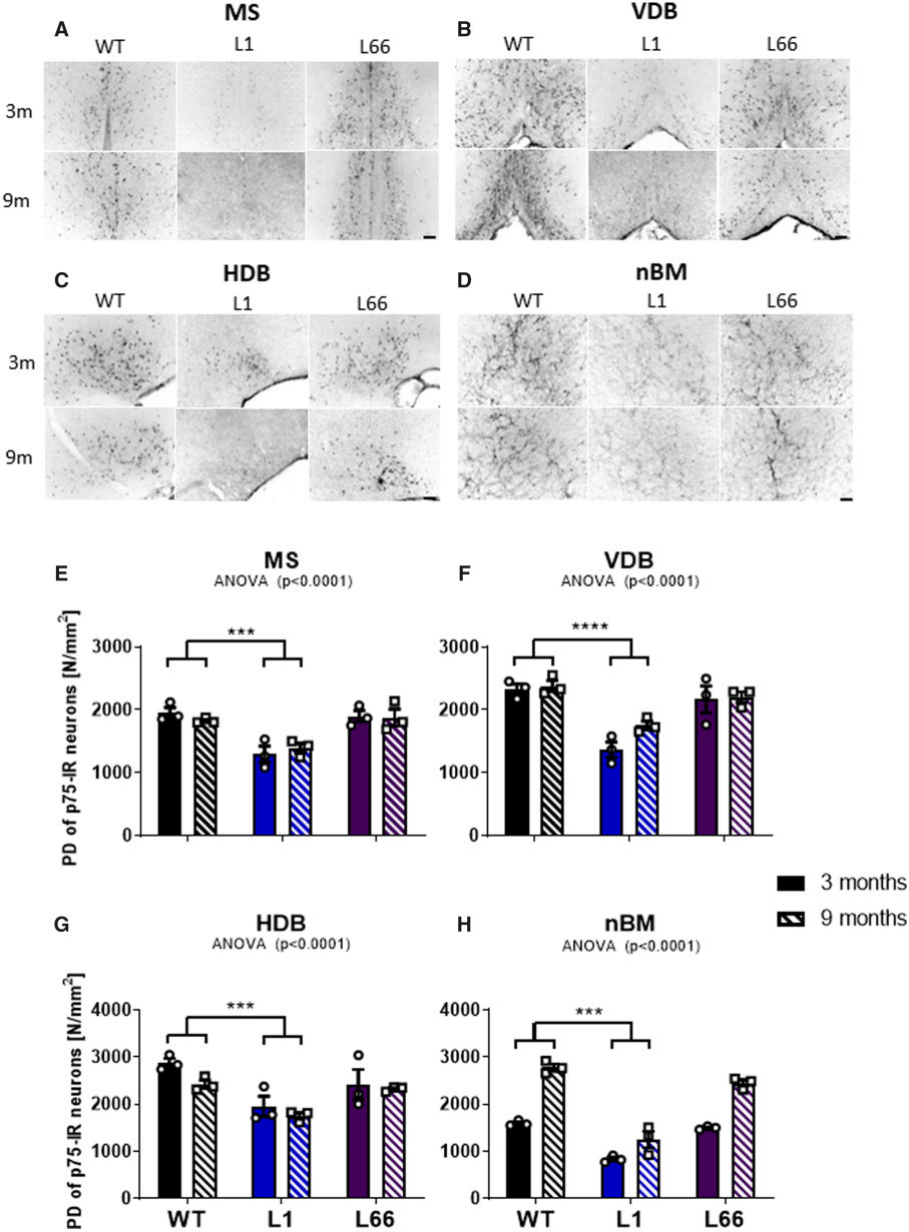
**p75^NTR^ immunohistochemistry in L1 and L66 basal forebrain.** Representative microscopic images of p75^NTR^ immunohistochemical staining and PD of p75-IR neurons (N/mm^2^) in the basal forebrain regions of (**A, E**) MS, (**B, F**) VDB, (**C, G**) HDB and (**D, H**) nBM in 3- and 9-month-old transgenic L1 (*n* = 3) and L66 (*n* = 3) and WT NMRI (*n* = 3) mice. Images were obtained at a 100× magnification. Scale bar represents 200 μm. The effect of genotype was determined using a two-way ANOVA, with transgene and age as factors followed by Newman–Keuls test. Data were considered statistically significant with alpha set to 5%. *P < 0.05, **P < 0.01 and ***P < 0.001. Data presented as mean ± SEM. PD = packing density.

The qualitative immune-histochemical examination of low-affinity neurotrophin receptor showed a decrease in both the number of p75^NTR^-IR neurons and the intensity of staining in L1 mice in MS, VDB, HDB and nBM in comparison to WT (Fig. 4A–D). This was already present at 3 months of age, and there was a further decline in mice at 9 months. In contrast, the p75^NTR^ staining of 3-month-old L66 mice showed a positive p75^NTR^-immunoreactivity resembling the pattern in WT, with particularly strong staining in cell bodies and neuropil. In addition, at 9 months, the intensity of p75^NTR^ staining did not seem to be altered in L66 or WT (Fig. 4).

The quantitative analysis revealed decreased packing density of p75^NTR^ in L1 mice as compared to WT in all studied structures (MS, *P* < 0.001; VDB, *P* < 0.001; HDB, *P* < 0.001; nBM, *P* < 0.001). In turn, age factor reached significance only in nBM. In 9-month-old animals of all groups, p75^NTR^ packing density was elevated as compared to 3 month-old mice and resembled the pattern seen in ChAT staining.

### Increased glial staining in both L1 and L66 basal forebrain

To gain further insight into the neuroinflammatory profile induced by tau overexpression in our Tg models, an analysis of Iba1 and GFAP-IR was conducted using immune-histochemical fluorescence staining methods. An initial focus was placed on the basal forebrain regions, for which a selective decline in ChAT positivity had been confirmed (see Figs 1 and 2). Areas included MS, nBM, VDB, HDB and ST.

**Figure 5:**
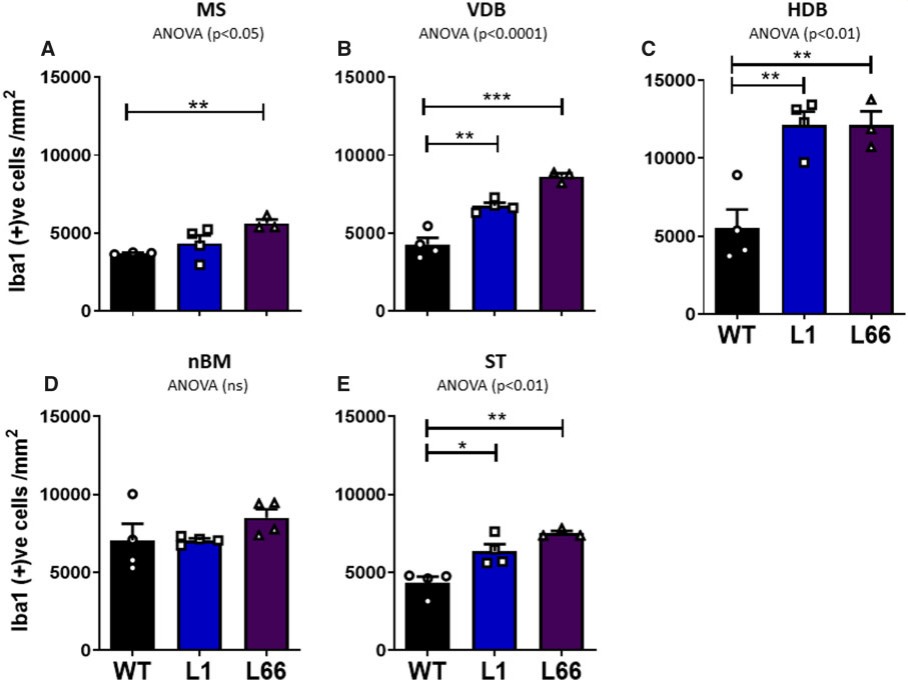
**Increased microglia in L1 and L66 mice.** Iba1 stereological cell-counting analysis from basal forebrain regions of the (**A**) MS, (**B**) VDB, (**C**) HDB, (**D**) nBM and (**E**) ST in transgenic L1 (*n* = 4), L66 (*n* = 4) and WT (NMRI, *n* = 4) mice at 6 months. Cell counts are expressed as the density of Iba1-positive cells (cells/mm^2^). Main effects of genotype were determined by one-way ANOVA, and the source of reliability was confirmed by *post hoc* Student’s *t*-test. Data were considered statistically significant when *P < 0.05, **P < 0.01 and ***P < 0.001. Data presented as mean ± SEM. ST = striatum.

**Figure 6:**
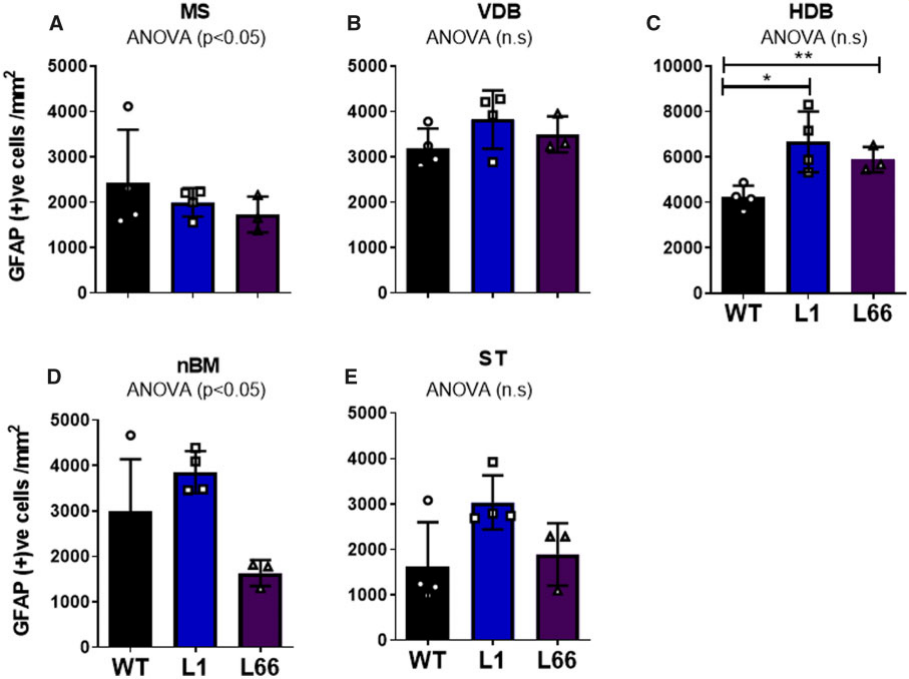
**Basal forebrain gliosis in both transgenic models.** GFAP stereological cell-counting analysis from basal forebrain regions of the (**A**) MS, (**B**) VDB, (**C**) HDB and (**D**) nBM; (**E**) ST, in transgenic L1 (*n* = 4), L66 (*n* = 4) and WT (NMRI, *n* = 4) mice at 6 months. Cell counts are expressed as the density of GFAP-positive cells (cells/mm^2^). Main effects of genotype were determined by one-way ANOVA, and the source of reliability was confirmed by *post hoc* Student’s *t*-test. Data were considered statistically significant when *P < 0.05, **P < 0.01 and ***P < 0.001. Data presented as mean ± SEM. ST = striatum.

A significant increase in the density of Iba1-IR was observed in L1 in VDB and HDB (*P* < 0.01) and ST (*P* < 0.05; Fig. 5B, C and E) at 6 months. The HDB was the only basal forebrain region, in which a significant increase in GFAP-IR was seen in L1 (*P* < 0.05, Fig. 6C, Supplementary Table 2). A robust increase in Iba1-positive microglia in L66 animals was observed for all basal forebrain regions apart from the nBM (*P* < 0.01; Fig. 5A–C and E). In parallel with L1, L66 also displayed a significant increase (*P* < 0.01) in the density of GFAP-reactive astrocytes within the HDB (Fig. 6, Supplementary Table 2). It is intriguing to note that, despite the robust cholinergic loss of ChAT in nBM of L1 animals (Figs 1 and 2; Supplementary Table 2), this was not matched by enhanced inflammatory responses of either Iba1 or GFAP.

### Increased glial immunostaining in hippocampal sub-regions of both L1 and L66

**Figure 7:**
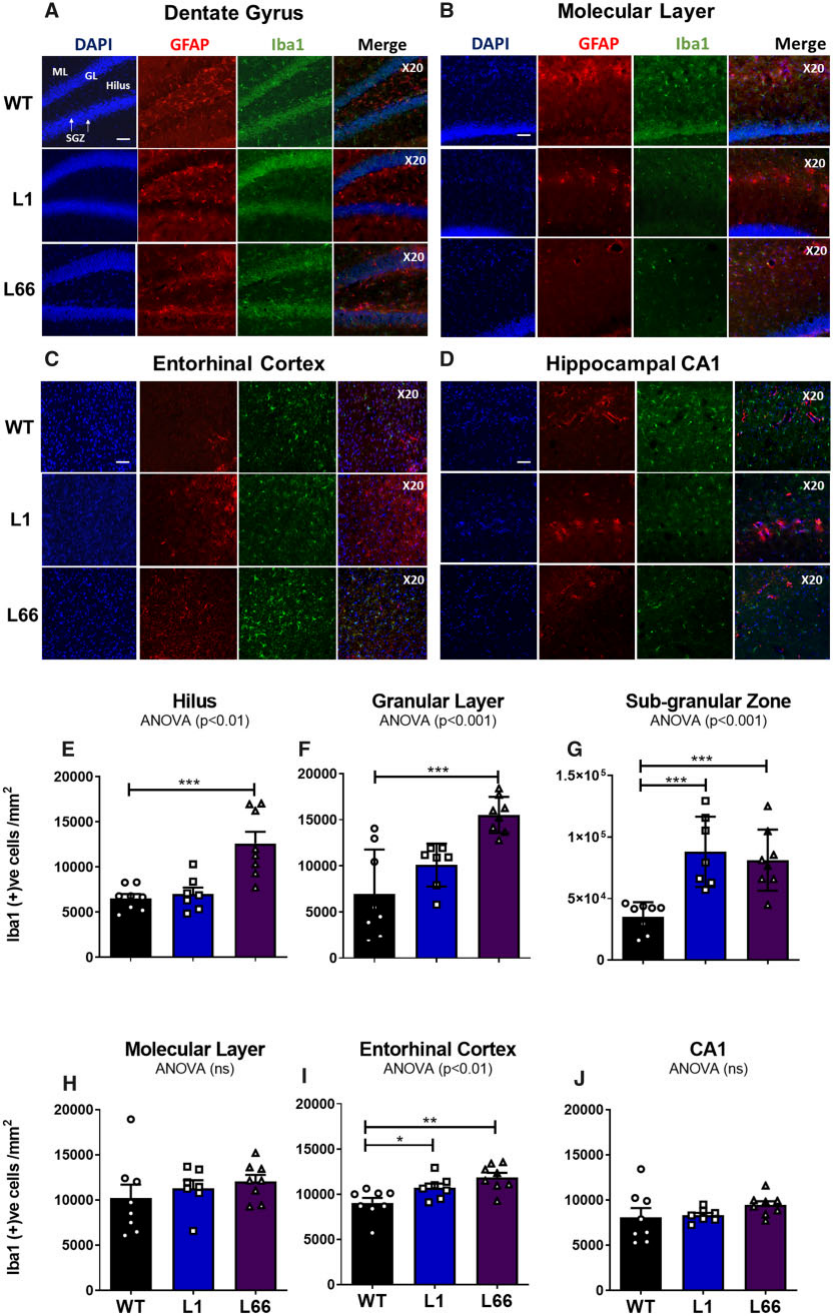
**Hippocampal neuroinflammation in L1 and L66 mice.** Representative fluorescence images for double labelling of microglia and astrocytes (**A–D**). Thirty micrometre sections are stained for Iba1 (green), GFAP (red) and 4′,6-diamidino-2-phenylindole (blue) for selected dentate gyrus sub-regions of the hilus, sub-granular zone, molecular layer, granular layer, hippocampal CA1 region and entorhinal cortex in WT mice AT 6 months. Representative images were obtained at 20× magnification. Scale bar, 55 µm. (E–J) Quantification of Iba1 stereological cell counting from dentate gyrus and hippocampal regions from transgenic L1 (*n* = 7), L66 (*n* = 8) and WT (NMRI, *n* = 8) mice at 6 months. Cell counts are expressed as Iba1-positive cells (cells/mm^2^). Quantification of GFAP stereological cell counts is found in Supplementary Fig. 2. Main effects of genotype for each area were determined using one-way ANOVA, and the source of significance was traced by *post hoc* Student’s *t*-tests. Data were considered statistically significant when *P < 0.05, **P < 0.01 and ***P < 0.001. Data presented as mean ± SEM. DAPI = 4′,6-diamidino-2-phenylindole.

To evaluate the microglial and astrocyte contribution to the disease pathogenesis induced by ChAT loss and tau overexpression in Tg models L1 and L66, immunohistochemical fluorescence staining methods were used on coronal mouse brain sections of the DG (hilus, molecular layer, granular layer, SGZ), hippocampus and EC. Representative images displaying selected brain regions for Iba1 and GFAP-IR counting in the hippocampus proper and DG micro-circuits are given in Fig. 7A and Supplementary Fig. 1. Robust microgliosis based on a significant increase in Iba1-IR density was observed in L1 mice and this was specific to the SGZ of the DG (SGZ *P* < 0.001; Fig. 7A, G, H) and the EC (*P* < 0.05; Fig. 7C and I). In L66, microgliosis was more widespread and heightened Iba1 reactivity was observed in the hilus of the DG, granular layer, SGZ (all *P*’s < 0.001), and the EC (*P* < 0.01), relative to WT mice (Fig. 7A, C-G, I).

No gliosis was observed in the molecular layer of the dentate or CA1 in either Tg line confirming the specificity of inflammatory responses to sub-regions. As for GFAP-IR astrocyte activation, there was a significantly increased density specific to the SGZ in L1 and L66 (*P* < 0.05), but not for other areas of the hippocampus **(**Fig. 7B, H, J, Supplementary Table 2**)**.

### Increased concentrations of IL-1β and C3 in the hippocampus of L1 and L66 mice

**Figure 8:**
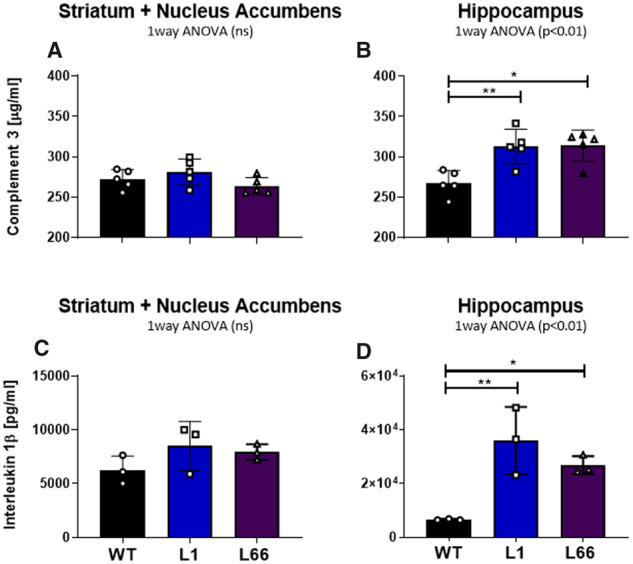
**Quantitative analysis of complement C3 in striatum with nucleus accumbens** (**A**), hippocampus (**B**) and interleukin 1β (**C**, **D**) from 6-month-old transgenic L1, L66 and WT (NMRI) mice at 6 months. For all genotypes, *n* = 3 for striatum and *n* = 5 for hippocampus. Concentrations were measured by enzyme-linked immunosorbent assay. Data are presented as mean ± SEM. Interactions between groups were determined using one-way ANOVA and the source of significance explored by *post hoc* Newman–Keuls test. **P < 0.01. Data presented as mean ± SEM.

Quantitative analysis of complement C3 and IL-1β was measured in ST with nucleus accumbens and hippocampus from 6-month-old Tg L1, L66 and WT (NMRI) mice (Fig. 8). In the ST, there was no difference in IL-1β and C3 concentrations between NMRI, L1 and L66 mice (Fig. 8A and C).

In the hippocampus, statistical analysis showed significantly higher concentrations of IL-1β and C3 in L1 (*P* < 0.01 for IL-1β and C3) and L66 mice (*P* < 0.05 for IL-1β and *P* < 0.01 for C3) compared to NMRI controls (Fig. 8B and D).

## Discussion

We report here on the distinct cholinergic and inflammatory phenotypes in the tau Tg mouse models L1 and L66, observations which permit a strong discrimination between Alzheimer’s disease-like and FTLD-like pathology in these mice. These models overexpress two different tau protein species: truncated paired helical filament-core tau (L1) and full-length mutant tau (L66) expressed under the pan neuronal-specific Thy1 promoter. In addition, it must be mentioned that L66 has an additional endoplasmic reticulum targeting signal sequence peptide, which is lacking in L1 mice (Melis *et al*, 2015a), albeit this is unlikely to have any influence on the following phenotypes reported in this study. Pathologically, mice mimic an Alzheimer’s disease-like pathology that follows a Braak-like progression (Braak and Braak, 1995) and features cognitive impairment (Melis *et al.*, 2015). L66 mice mimic FTLD-like pathology and feature an early-onset motor deficit that becomes more severe with age (Melis *et al.*, 2015).

### L1 exhibits features of Alzheimer’s disease

In the basal forebrain of L1, we observed a significant loss of cholinergic neurons at 3, 6 and 9 months, compared to control groups, in two independently conducted studies. Lack of reproducibility between rodent pathological pre-clinical studies is a prevalent issue (Treuting *et al.*, 2016; Ward *et al.*, 2017). Therefore, these two independent, parallel studies robustly support this cholinergic phenotype observed in L1 and lack thereof in L66.

In agreement with the Alzheimer’s disease-like phenotype of L1, the significant basal forebrain cholinergic neuron loss matches that observed in post-mortem brains of patients with Alzheimer’s disease (Davies and Maloney, 1976; Whitehouse *et al.*, 1982). Cholinergic afferents of the MS and VDB project towards the hippocampus via the septo-hippocampal pathway, and cholinergic somata in the nBM primarily target the cortical neurons via the baso-cortical pathways (Khakpai *et al.*, 2013). Specifically, cholinergic afferents from the MS to hippocampus appear crucial in the regulation of spatial and mnemonic function (Okada *et al.*, 2015). A lowering in basal forebrain cholinergic ChAT-IR neurons would therefore contribute to an explanation of the behavioural age-dependent deficits in episodic-like memory previously observed in L1 (Melis *et al.*, 2015) and is at the same time reminiscent of the pathology and cognitive decline typically at play in Alzheimer’s disease (Grothe *et al.*, 2010; Pedroso *et al.*, 2018).

A reduction in striatal ChAT was also observed in L1 at 3, 6 and 9 months, data that match that of the human Alzheimer’s disease brain, where there is a reduction in ChAT in the ventral and dorsal ST (Boissière *et al.*, 1997). In addition, a significant reduction in AChE staining intensity was observed in both cortex and hippocampus of L1 at 3 and 12 months.

In line with the ChAT reduction in L1, hippocampal AChE levels have been reported to be significantly reduced in patients with mild cognitive impairment and early Alzheimer’s disease (Rinne *et al.*, 2003), displaying robust correlations to cognitive impairment (Shinotoh *et al.*, 2000; Bohnen *et al.*, 2005). Given the cognitive phenotype in L1 (Melis *et al.*, 2015), the observed reduction in AChE levels may explain some of the spatial learning and memory deficits known for L1 mice.

Survival of basal forebrain cholinergic neurons is entirely dependent on the binding of nerve growth factor to hippocampal and cortical receptors including tropomyosin receptor kinase A and p75^NTR^ (Niewiadomska *et al.*, 2011). An age-dependent reduction in p75^NTR^ was observed in L1 from 3 months onwards in basal forebrain regions of the MS, VDB, HDB and nBM. These parallel post-mortem observations in patients with Alzheimer’s disease, in which p75^NTR^ is significantly reduced in the nucleus basalis of Meynert, the equivalent of the murine nBM (Salehi *et al.*, 2000; Mufson *et al.*, 2002). However, no age-dependent reduction in ChAT was observed in L1 mice suggesting that ChAT reduction occurs early on and this pathology remains steady with age.

At the same time, an increase in microglial activation in the basal forebrain of L1 mice may also be initiated by a ChAT reduction at 6 months or earlier. Equivalent studies in rodent models have shown that selective lesioning of the basal forebrain leads to a direct increase in susceptibility to cognitive deficits induced by acute inflammation (Field *et al*, 2012); therefore, this increase in microglial activation may also contribute to an explanation of the cognitive deficits observed in L1 at 3 months of age (Melis *et al.*, 2015). Enhanced neuroinflammation was confirmed in L1 as a significant increase in the expression of Iba1 in the VDB, HDB and ST. Our data suggest that microglial activation in L1 may follow a cholinergic loss in corresponding basal forebrain regions, albeit microglial activation was observed at 6 months only, and was not investigated at 3 months in this study. However, this could suggest a potential predisposition of regions affected by cholinergic neuron loss, as being susceptible to microglial recruitment and activation.

This hypothesis has been explored previously in mice, where pre-existing cholinergic deficits can result in a predisposition towards inflammatory insult, through up-regulation of inflammatory markers IL-1β, tumour necrosis factor alpha and IL-6, following selective basal forebrain lesioning (Field *et al.*, 2012). A significant increase in microglial marker Iba1 was observed across hippocampus, EC, of L1 mice. Of note is the particularly high expression of microglia in the SGZ, as this specific area of the DG is where neurogenesis primarily occurs (van Praag *et al.*, 2002). Previous studies have suggested that a disruption in neurogenesis, particularly through neuroproliferation observed in the DG, may be potentially associated with the age-dependent decrease in hippocampal learning and memory in Alzheimer’s disease (Jin *et al.*, 2004). Specifically, microglia and astrocytes influence neurogenesis through the induction of apoptosis and the negative regulation of neurogenesis via Notch-mediated signalling pathways (Sierra *et al.*, 2010; Wilhelmsson *et al.*, 2012). Although not established in this study, it is conceivable that both microglia and astrocyte activation will compromise neurogenesis, thereby explaining some of the cognitive deficits observed in L1/L66 from 3 months of age (Melis *et al.*, 2015a). In addition, a significant increase in IL-1β, a marker of activated microglia, was observed in the hippocampus of L1 mice at 6 months. Heightened expression of pro-inflammatory cytokines, such as IL-1β, has been associated previously with mild cognitive impairment in the brains of patients with Alzheimer’s disease (Forlenza *et al.*, 2009). This may be a therapeutic target as the reduction in IL-Iβ attenuated tau pathology in 3×Tg-AD mice (Kitazawa *et al.*, 2011).

Less overlap was observed between the loss of acetylcholine in hippocampus and gliosis. Since cholinergic afferents from the basal forebrain project directly to the hippocampus (Perez-Lloret and Barrantes, 2016), loss of cholinergic labelling in basal forebrain neurons was expected to evoke a secondary inflammatory response in the hippocampus in L1, but not necessarily in L66. The fact that L66 has no cholinergic phenotype in the MS, but expressed strong astrogliosis in the hippocampus, is therefore intriguing. Previously, it has been shown that hippocampal astrocytes respond to acetylcholine released from synaptic terminals, suggesting the possible existence of cholinergic synapse–astrocyte interactions (Araque *et al.*, 2002). The data reported here seem to suggest a dissociation between cholinergic loss in basal forebrain and inflammatory responses in their target structures. We confirmed that the lack of Iba1 microglial activation does not preclude an up-regulation of other neuroinflammatory markers, especially IL-1β and complement C3.

Previous studies have also shown that levels of microglia are substantially elevated in the brains of ageing rodents, and this elevation is not necessarily associated with a cholinergic deficit, particularly within the MS and VDB (McQuail *et al.*, 2011). Furthermore, no differences in Iba1 or GFAP morphology were observed in either basal forebrain or hippocampus of L1 or L66 mice at 6 months. Overall, this suggests that the cholinergic system and inflammation may coincide for specific basal forebrain regions, but that they do not appear to overlap necessarily in region-specific targets in the above models.

### L66 is more FTLD-like

In contrast to L1 mice, L66 exhibited no difference in total ChAT-IR neuron cell count at 3–9 months in the basal forebrain (apart from Broca’s band) suggesting relatively intact cholinergic function. This finding supports the proposed FTLD-like phenotype for these mice (Melis *et al.*, 2015) and matches the data from patients with FTLD, in which no significant difference in ChAT activity, relative to age-matched controls, was reported (Procter *et al.*, 1999; Bowen *et al.*, 2008, Hirano *et al.*, 2010). Second, a significant reduction in cortical AChE was observed as late as 12 months in L66, with no difference observed in the hippocampus at the same age. Again, this is reminiscent of patients with FTLD exhibiting no differences in hippocampal AChE levels (Hirano *et al*., 2006). Third, no changes in p75^NTR^ staining were observed in L66 models at 3 or 9 months, suggesting that neither neuronal cell loss nor loss of cholinergic function accounts for the motor phenotype or intact spatial learning phenotype previously observed in these mice (Melis *et al.*, 2015). The above findings therefore strongly translate to the disease phenotype of FTLD in L66 mice.

In L66, a robust microglial activation was also observed in basal forebrain and ST. This result is interesting, as none of these regions exhibited any change in cholinergic neuron density through the measurement of ChAT activity. The pathway of inflammation in FTLD is much less understood than in Alzheimer’s disease, although studies in patients with FTLD have revealed an increase in pro-inflammatory cytokines including tumour necrosis factor alpha and tumour necrosis factor-β (Sjögren *et al.*, 2004). In the brain of patients with FTLD, microglial activation has been widely observed, using immunohistochemistry, in frontal and temporal cortex (Lant *et al.*, 2014), and in dorsolateral prefrontal cortex and hippocampus (Cagnin *et al.*, 2004).

Our study provides the first indication that microglial activation in tau Tg L66 mice that mimic FTLD may be widespread and related to regions affected by tau deposition, such as frontal cortex, EC and hippocampus (Melis *et al.*, 2015) rather than cholinergic degeneration. Activated microglia could promote pro-inflammatory activation of astrocytes (Lian *et al.*, 2016). Interleukin-1α and IL-1β, microglia-derived acute phase cytokines, and activated astrocytes are known to up-regulate the expression of the astrocyte-derived complement protein C3 (Maranto *et al.*, 2008). These cytokines form complex interactions that may be capable of self-propagation, thus leading to chronic overexpression of glial cytokines with neurodegenerative consequences (Mrak *et al.*, 1995). Self-propagation may be facilitated by means of several reinforcing feedback loops. For instance, microglia express the C3a receptor (Zhang *et al.*, 2014) and their function could be impacted by astrocytic release of C3. Directly activated microglia release IL-1 and stimulate the astrocyte complement system, leading to additional microglial activation through C3a receptors. Thus, a microglial IL-1β-astrocytic C3–microglial C3a receptor positive feedback loop could govern inflammatory dynamics and tau neuropathology. These interactions with tau remain poorly understood, but available data suggest that the severity of both astrogliosis and microglial activation is linked to tau species and to the severity of aggregated tau.

Finally, both L1 and L66 show increased GFAP- and Iba1-IR cells in the basal forebrain HDB and SGZ of the DG. In FTLD, astrocytosis has been reported in the absence of neuronal loss (Broe *et al.*, 2004), whilst astrocytic pathology in Alzheimer’s disease is directly associated in regions exhibiting neuronal loss (Forman *et al.*, 2005).

## Summary

Collectively, two independent studies have observed significant loss of cholinergic staining in the basal forebrain in L1, whilst there is also a sub-regional microglial activation. These observations may help explain in part the cognitive deficits observed in L1 and confirm the Alzheimer’s disease-like phenotype. By contrast, L66 exhibited a relatively intact cholinergic system but presented with a robust and widespread microglial activation uncorrelated with cholinergic pathology.

Given the differences in the human tau species expressed in these two mouse models, the overexpression of two different forms of tau can lead to two very distinct and robust phenotypes that show characteristic features of Alzheimer’s disease-like and FTLD-like tauopathies. Not only does L1 mimic an age-dependent, Braak-like spread of tau pathology, it also features a loss of cholinergic function that is characteristic of Alzheimer’s disease. This differs from L66, with its more robust and widespread tau pathology with considerably more overlap of inflammation, that make it representative of FTLD.

## Supplementary Material

fcaa033_Supplementary_Data

## Abbreviations

AChE =: acetylcholinesterase
BSA =: bovine serum albumin
C3 =: complement C3 (astrocytic)
ChAT =: choline acetyltransferase
DG =: dentate gyrus
EC =: entorhinal cortex
FTLD =: frontotemporal lobar degeneration
GFAP =: glial fibrillary acidic protein
HDB =: horizontal limb of the diagonal band of Broca
Iba1 =: ionized calcium binding adaptor molecule 1
Il-1β =: interleukin-1 beta
L1 =: Line 1; L66 = Line 66
MS =: medial septum
nBM =: nucleus basalis magnocellularis
NFT =: neurofibrillary tangle
p75^NTR^ =: p75 neurotrophin receptor
RT =: room temperature
SGZ =: sub-granular zone
ST =: striatum
Tg =: transgenic
VDB =: vertical limb of the diagonal band of Broca
WT =: wild type
