# Supplementary material for: Cholinergic and inflammatory phenotypes in transgenic tau mouse models of Alzheimer’s disease and frontotemporal lobar degeneration
Source: Brain Commun. 2020 Mar 30;2(1):fcaa033. doi: 10.1093/braincomms/fcaa033 (PMC7425524; doi:10.1093/braincomms/fcaa033)
Supplement: fcaa033_Supplementary_Data [file fcaa033_supplementary_data.pdf]

Supplementary Data

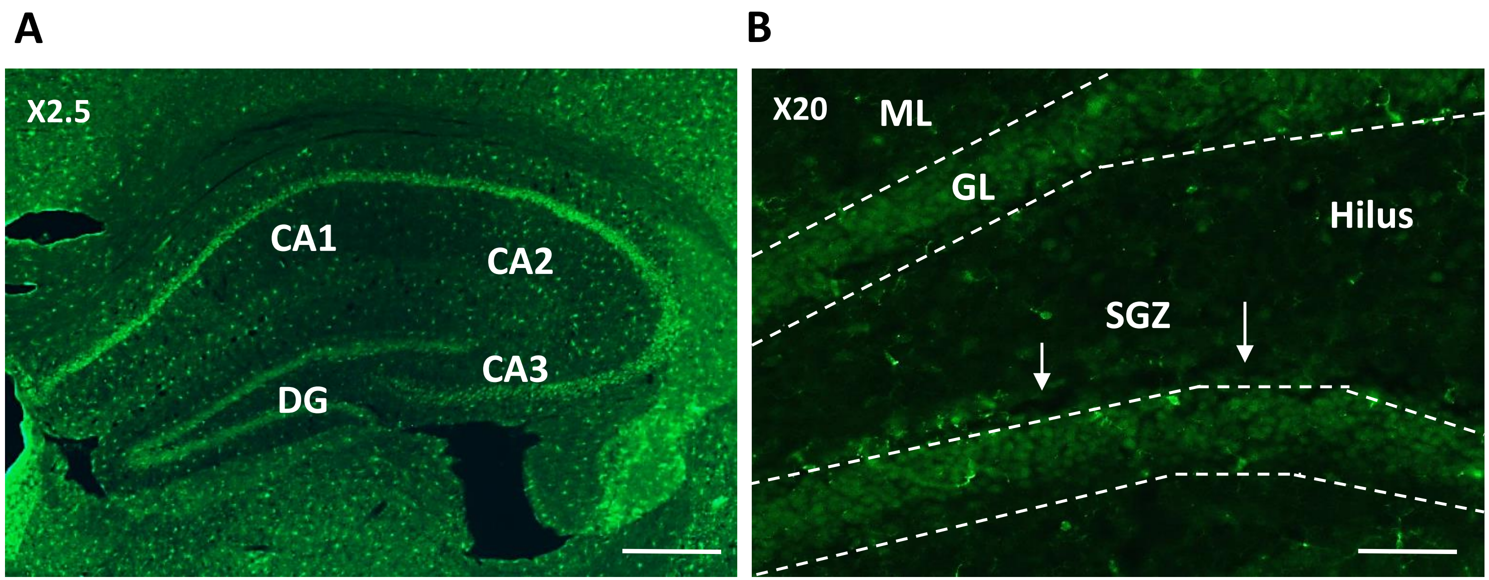

Supplementary Figure 1. Microglial staining in the hippocampus. Representative fluorescence images stained for Iba1 (green) for selected hippocampal / dentate gyrus **(A)** sub-regions of the hilus, subgranular zone (SGZ), granular layer (GL) and molecular layer (ML) **(B)** in a WT NMRI mouse aged 6 months. Representative images were obtained at X2.5 and X20 magnification, respectively. Scale bar, 55µm.

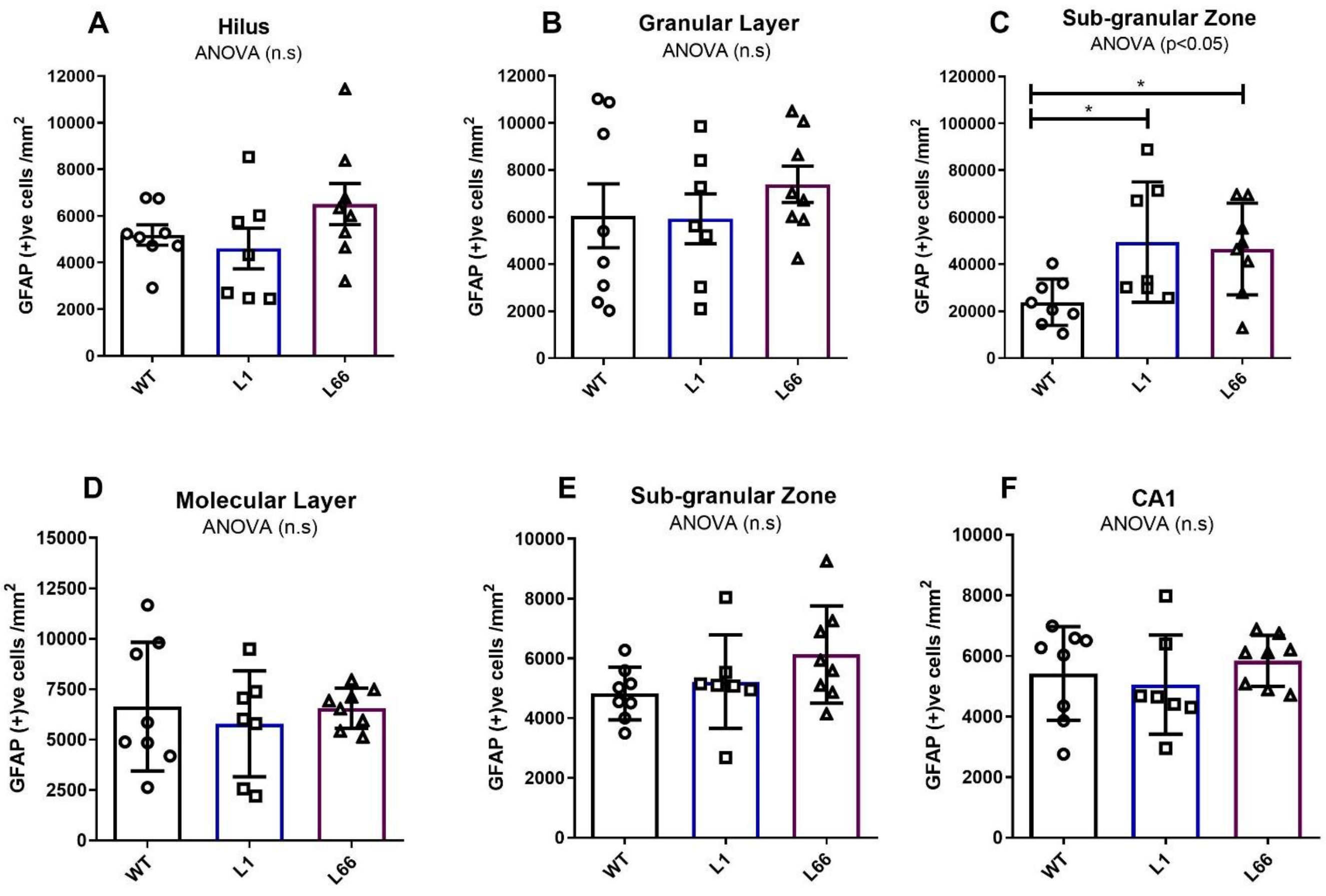

Supplementary Figure 2. Astrocyte populations in the hippocampus of L1 and L66 mice. Quantification of GFAP stereological cell-counting from dentate gyrus and hippocampal regions from transgenic L1 (n=7), L66 (n=8) and WT (NMRI, n=8) mice at 6 months. Cell counts are expressed as Iba1-positive cells (cells mm^{-2}). Main effects of genotype for each area was determined using 1-way ANOVA, and the source of significance was traced by post-hoc Student t-tests. Data was considered statistically significant when * $p<0.05$ and data are presented as mean +SE.

Supplementary Table 1. Antibody specifications. Summary of primary and secondary antibodies used in this study, including host species, dilution and supplier. n/a, not applicable.

1° Antibody	Immunogen/ epitope	Host Species	Dilution	Mono/ polyclonal	Supplier	2° Antibody	Dilution	Supplier
**ChAT (AB144P)**	Human placental anti-choline acetyltransferase	Goat IgG	1/100	Polyclonal	Merck Millipore	Donkey α Goat (Alexa Fluor® 488; ab150129); Donkey α Goat H+L conjugate (SA-5014)	1/100; 1/5000	Abcam; Vector Laboratories Ltd.
**$p75^{NTR}$ (MA1-18401)**	Extracellular domain of human $p75^{NTR}$	Rabbit IgG	1/100	Monoclonal	Fisher Scientific UK	Goat α Mouse Peroxidase Conjugate H+L (AP124P0)	1/100	Vector Laboratories Ltd.
**Iba1 (NCNP24)**	Iba1 (Ionized calcium binding adaptor molecule 1) C-terminal	Rabbit IgG	1/300	Monoclonal	Alpha Laboratories Ltd.	Goat α Rabbit (Alexa Fluor® 488; A11008))	1/100	Fisher Scientific UK
**GFAP [GF5] (ab10062)**	Murine ant-GFAP (Glial fibrillary acidic protein)	Mouse IgG2b	1/300	Monoclonal	Abcam	Goat α Mouse (Alexa Fluor® 594; A11005)	1/200	Fisher Scientific UK

Supplementary Table 2. ChAT, Iba1 and GFAP statistical analysis in the basal forebrain and hippocampus. Data represents stereological cell-counting analysis in female L1 (n=4) and L66 (n=4) mice compared with WT (NMRI, n=4) mice; all aged 6 months. Note that a ChAT 3-9 months comparison is not included in this summary, so as to allow 6 months comparisons for Iba1 and GFAP in animals of the same cohort). ↑↑↑ indicates significance level of $p<0.001$, ↑↑ = $p<0.01$ and ↑ = $p<0.05$ (Student's t-test), and (-) indicates parameter not measured. Direction of arrow indicates increase (↑) or decrease (↓) relative to WT (NMRI) mice.

	Iba1 + ve cells/mm^2		GFAP + ve cells/mm^2		Total ChAT + ve neurons	
**Brain Sub-Regions:**	**L1 vs. WT**	**L66 vs. WT**	**L1 vs. WT**	**L66 vs. WT**	**L1 vs. WT**	**L66 vs. WT**
**Hilus (DG)**	p=0.5669	↑↑↑	p=0.5458	p=0.2008	-	-
**Granular Layer (DG)**	p=0.1432	↑↑↑	p=0.9443	p=0.4062	-	-
**Sub-granular Zone (DG)**	↑↑↑	↑↑↑	↑	↑	-	-
**Molecular Layer (DG)**	p=0.5583	p=0.2912	p=0.5847	p=0.9485	-	-
**Entorhinal Cortex**	↑	↑↑	p=0.5519	(↑) p=0.0663	-	-
**Hippocampus (CA1)**	p=0.8604	p=0.2391	p=0.6625	p=0.5510	-	-
**Medial Septum (MS)**	p=0.3441	↑↑	p=0.4941	p=0.3670	↓	p=0.7777
**Nucleus Basalis Magnocellularis (nBM)**	p=0.9934	p=0.2692	p=0.2130	p=0.1091	↓↓	(↓) p=0.0796
**Vertical Limb of the Diagonal Band of Broca (VDB)**	↑↑	↑↑↑	p=0.1552	p=0.3882	↓↓	p=0.5171
**Horizontal Limb of the Diagonal Band of Broca (HDB)**	↑↑	↑↑	↑	↑↑	↓	p=0.3792
**Striatum (ST)**	↑	↑↑	(↑) p=0.0571	p=0.3676	↓↓	p=0.2946
**Nucleus Accumbens (NAcc)**	↑	↑↑	p=0.1193	p=0.6175	p=0.5052	p=0.3197
